# Acupuncture treatment on the motor area of the scalp for motor dysfunction in patients with ischemic stroke: study protocol for a randomized controlled trial

**DOI:** 10.1186/s13063-017-2000-x

**Published:** 2017-06-20

**Authors:** Jun Wang, Jian Pei, Dhiaedin Khiati, Qinhui Fu, Xiao Cui, Yi Song, Minghang Yan, Lijun Shi, Yiwen Cai, Yuhong Ma

**Affiliations:** 1 Department of Acupuncture, Longhua Hospital, Shanghai University of Traditional Chinese Medicine, No. 725 South WanPing Road, XuHui District, Shanghai, 200032 China; 2 Department of Rehabilitation, Shanghai Changning District Tianshan Hospital of Traditional Chinese Medicine, No. 868 Loushanguan Road, Changning District, Shanghai, 20005l China; 3 School of Medicine, Royal College of Surgeons in Ireland - Medical University of Bahrain, 15503 Adilya, Bahrain

**Keywords:** Ischemic stroke, Motor dysfunction, Jiao’s scalp acupuncture, Study protocol

## Abstract

**Background:**

Scalp acupuncture has shown a remarkable treatment efficacy on motor dysfunction in patients with stroke in China, especially the motor area of Jiao’s scalp acupuncture, which is the most widely used treatment. However, previous studies have summarized that the clinical curative effect of acupuncture treatment for stroke remains uncertain. Meanwhile, no randomized controlled trials on Jiao’s scalp acupuncture have been performed. The aim of this study is to evaluate the efficacy and safety of Jiao’s scalp acupuncture for motor dysfunction in ischemic stroke.

**Methods/design:**

This is an assessor- and analyst-blinded, randomized controlled trial. One hundred and eight stroke patients with motor dysfunction meeting the inclusion criteria will be allocated by a 1:1 ratio into either an acupuncture treatment group or a control group. Stroke patients in the control group will receive conventional rehabilitation treatment, whereas a combination of Jiao’s scalp acupuncture and conventional rehabilitation treatment will be applied to the acupuncture group. Forty treatment sessions will be performed over an 8-week period. The Fugl-Meyer Assessment scale will be assessed as the primary outcome measure. The Modified Barthel Index, the Stroke-Specific Quality of Life, and the Stroke Syndrome of Traditional Chinese Medicine scales will be selected as secondary outcome measurements. All assessments will be conducted at baseline, week 4 (treatment 20), week 8 (treatment 40), week 12 (follow-up), and week 16 (follow-up).

**Discussion:**

This is the first trial evaluating the efficacy and safety of Jiao’s scalp acupuncture for motor dysfunction in ischemic stroke. The results of this trial are expected to provide relevant evidence demonstrating that Jiao’s scalp acupuncture can be used as an effective rehabilitation treatment method for improving motor dysfunction in ischemic stroke.

**Trial registration:**

ClinicalTrials.gov, NCT02871453. Registered on 17 July 2016.

**Electronic supplementary material:**

The online version of this article (doi:10.1186/s13063-017-2000-x) contains supplementary material, which is available to authorized users.

## Background

The epidemic survey data from the Global Burden of Diseases, Injuries, and Risk Factors Study (GBD 2010) ranked stroke as the second most common cause of death, the most common cause of disability [1], and the third most common cause of disability-adjusted life years (DALYs) worldwide [2]. Over the past two decades, the absolute number of people with first stroke (16.9 million), stroke survivors (33 million), and stroke-related deaths (5.9 million) and the overall global burden of stroke (DALYs lost, 102 million) have been on the rise [3]. Statistics from the American Heart Association show that approximately 795,000 people experience a new or recurrent stroke each year [4]. In China, stroke is reported as one of the most common causes of death in both urban and rural areas [5, 6]. An epidemiologic study published in 2007 indicates that China has more than 7 million stroke survivors. Ischemic stroke is the most common subtype of stroke, accounting for about 80% of all strokes. Approximately 70% of stroke survivors experience functional disabilities, motor dysfunction being the most significant symptom [7, 8]. The activities of daily living and social participation are limited in patients with stroke due to motor dysfunction; this greatly influences the patient’s quality of life and return to society. Also, these limitations place a heavy burden on the family and society as a whole, becoming a huge public health problem [9].

Western conventional treatment of patients with stroke includes pharmacological treatments, surgical operation, and multi-professional rehabilitation. This approach is a multi-disciplinary and complex procedure designed to improve functional disability, prevent complications, and reduce the risk of additional attacks at any stage of stroke [10, 11]. In China, stroke rehabilitation mainly develops the clinical mode of combining traditional Chinese medicine and western medicine. Acupuncture use as a complementary or alternative therapy has increased worldwide and has become widely applied to stroke rehabilitation over the last decade [12, 13], which confirms that the efficacy of acupuncture can have a great impact on stroke management [14]. As early as 1997, the National Institutes of Health (NIH) panel recommended acupuncture as a complementary rehabilitation treatment for stroke [15]. In 2002, the World Health Organization (WHO) also recommended acupuncture as a treatment for stroke; they thought that acupuncture treatment could improve a variety of functional disabilities such as motor, sensation, speech, and other neurologic functions [16].

Many studies [17] have shown that scalp acupuncture has a remarkable treatment efficacy on motor dysfunction in stroke patients in China, and in recent years, various scalp acupuncture schools have been developed to treat motor dysfunction in stroke patients in hospitals. The motor area of Jiao’s scalp acupuncture or the anterior oblique line of the vertex-tempora of the international standardized scalp partition is usually selected as the scalp acupuncture stimulatory region to treat motor dysfunction in stroke patients; however, Jiao’s scalp acupuncture is the more widely used of the two techniques [18]. Jiao’s scalp acupuncture combines a modern understanding of neuroanatomy and neurophysiology with traditional techniques of Chinese acupuncture to develop a radical new tool for affecting the functions of the central nervous system and accepts a central theory that incorporates brain functions into Chinese medicine principles. The motor area of Jiao’s scalp acupuncture that is specifically used for treatment of motor dysfunction after stroke is equivalent to the structure of the precentral gyrus of the cerebral cortex on the scalp projection [19]. However, no randomized controlled trials (RCTs) have been performed to demonstrate the clinical curative effect of the motor area of Jiao’s scalp acupuncture treatment on motor dysfunction in patients with stroke.

Moreover, with the rapid development of evidence-based medicine, any clinical intervention strategies which are used must be backed by a high level of evidence-based support. Although acupuncture as a treatment for stroke has become widely accepted and has shown a better clinical curative effect than conventional treatments, numerous meta-analysis reviews [14, 20–25] based on clinical RCTs have summarized that the clinical curative effect of acupuncture treatment in stroke remains uncertain. The reason is the often low quality of the available trials; further large-scale RCTs of better quality are still needed. Furthermore, system reviews [26, 27] based on scalp acupuncture treatment of motor dysfunction in stroke concluded that the evidence was insufficient to warrant a clinical recommendation due to the generally low methodological quality of the included studies. In addition, there was no evidence available on the safety of this treatment because none of the trials reported adverse effects.

At the same time, almost all the RCTs used the evaluation system of modern medicine, and based on the evaluation system of modern medicine, the clinical curative effect of acupuncture treatment for stroke has some limitations. For example, many motor function assessment scales do not accurately reflect the actual curative effect of acupuncture based on the syndrome differentiation of traditional Chinese medicine (TCM) in a certain stage of stroke. Thus, we need to use TCM scales based on TCM symptomatology to effectively evaluate the curative effect of acupuncture. It is worth considering the best way to solve the issue of how to apply standardized RCTs to accurately evaluate the individualized curative effect of acupuncture treatment based on the theory of TCM in patients with stroke.

Based on the above, the purpose of this study is to observe the therapeutic effect of scalp acupuncture using Jiao's motor area for motor dysfunction in ischemic stroke patients by using both international general evaluation scales and the TCM evaluation system.

## Methods/design

### Objectives

The objective of this proposed study is to investigate whether Jiao's scalp acupuncture treatment could improve significantly motor function in patients with ischemic stroke.

### Study design

**Fig. 1:**
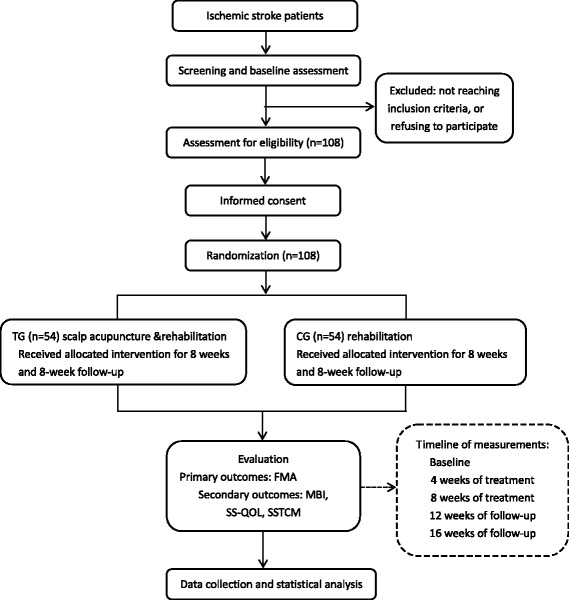
Consolidated Standards of Reporting Trials (CONSORT) flow diagram showing subject allocation to the study conditions (TG, treatment group; CG, control group; FMA, Fugl-Meyer Assessment; MBI, Modified Barthel Index; SS-QOL, Stroke-Specific Quality of Life Scale; SSTCM, Stroke Syndrome of Traditional Chinese Medicine)

**Fig. 2:**
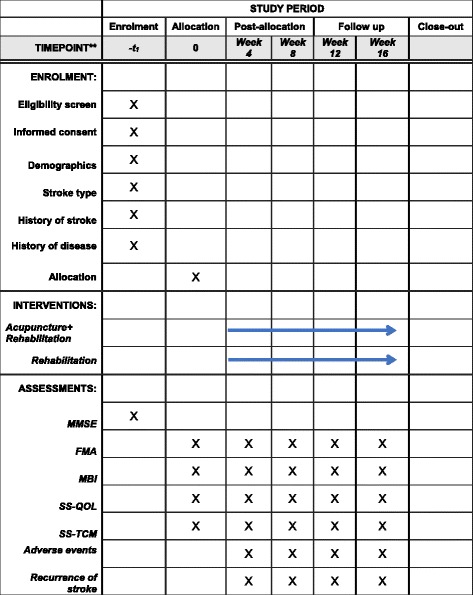
SPIRIT figure

This is an outcome assessor- and data analyst-blinded, randomized controlled study. The study is planned to be conducted from 1 January 2015 to 31 December 2016 in Longhua Hospital affiliated with the Shanghai University of Traditional Chinese Medicine. Stroke patients with motor dysfunction meeting the inclusion criteria will be allocated by a 1:1 ratio into either an acupuncture treatment group or a control group. Stroke patients in the control group will receive conventional rehabilitation treatment, whereas a combination of Jao’s scalp acupuncture and conventional rehabilitation treatment will be applied to the acupuncture group. The Fugl-Meyer Assessment (FMA) scale will be assessed as the primary outcome measure. The Modified Barthel Index (MBI), the Stroke-Specific Quality of Life (SS-QOL), and the Stroke Syndrome of Traditional Chinese Medicine (SSTCM) scales will be selected as secondary outcome measurements. All assessments will be conducted at baseline, week 4 (treatment 20), week 8 (treatment 40), week 12 (follow-up), and week 16 (follow-up). Figure 1 summarizes the flow of the entire trial. Figure 2 shows the study timeline, according to the Standard Protocol Items: Recommendations for Interventional Trials (SPIRIT) diagram. Additional file 1 presents the SPIRIT checklist.

### Inclusion criteria

Participants meeting the following inclusion criteria will be included: (1) Stroke patients between 40 and 70 years old; (2) stroke was diagnosed according to the criteria of cerebral arterial thrombosis in western medicine, and apoplexy in Chinese medicine; (3) ischemic stroke confirmed by a brain computed tomography (CT) or magnetic resonance imaging (MRI) scan, where patient has a stable medical condition and clear awareness; (4) patient experienced a recent stroke, between 1 month to 6 months after onset; (5) stroke with limb motor dysfunction; (6) sufficient cognition to follow commands and Mini-Mental State Examination (MMSE) score >24; (7) voluntary participation and informed consent signed.

### Exclusion criteria

Participants with any of the following exclusion criteria will be excluded: (1) stroke with conscious disturbance or serious cognitive impairment; (2) presence of another chronic disorder, including severe Parkinson’s disease, cardiac disease, cancers, epilepsy, or chronic alcoholism; (3) impaired hepatic or renal function; (4) bleeding tendencies; (5) oversensitivity to acupuncture; (6) participation in another clinical trial.

### Informed consent

Prior to the study, the general study process will be explained at participant recruitment. Participants will be informed that participation in the trial is absolutely voluntary and that they can withdraw from the trial at any time. In the event of their withdrawal, study data collected on the participant will not be deleted and will be used in the final analyses. Written informed consent will be obtained from each participant before they undergo any interventions related to the study.

### Interventions

The study is a randomized clinical trial carried out in the inpatient and outpatient rehabilitation departments of two hospitals. A total of 108 patients with ischemic stroke will be recruited. The patients will be randomly assigned to two different groups: (1) the treatment group and (2) the control group. The treatment group (*n* = 54) will receive Jiao’s scalp acupuncture combined with rehabilitation treatment five times per week for 8 weeks, and the control group (*n* = 54) will receive rehabilitation treatment five times per week for 8 weeks. Both groups will be evaluated at baseline, week 4 (treatment 20), week 8 (treatment 40) week, week 12 (follow-up), and week 16 (follow-up). Both groups will receive conventional stroke rehabilitation treatment during the whole 8-week study period. The rehabilitation program was designed according to the Chinese stroke rehabilitation treatment guidelines, which include physical therapy (PT) and occupational therapy (OT) for 5 days a week [28]. Western medicine will be permitted for conventional symptomatic treatment (e.g., antihypertensive drugs, drugs to regulate blood sugar, lipid-lowering drugs, and drugs to inhibit platelet aggregation). Chinese herbal medicine and Chinese patent drugs will be prohibited during the trial.

### Scalp acupuncture treatment

The acupuncture intervention complies with the Standards for Reporting Interventions in Clinical Trials of Acupuncture (STRICTA) guidelines. Moreover, all the acupuncturists will receive special training to achieve a sound understanding of the acupuncture intervention and to normalize the practices across different acupuncturists. The trial adheres to the STRICTA guidelines [29, 30].

The parameters for scalp acupuncture are set as follows:

1. Location of the motor area of Jiao’s scalp acupuncture: This area is located over the anterior central convolution of the cerebral cortex. It is a line starting from a point (known as the upper point of the motor area) 0.5 cm posterior to the midpoint of the anterior-posterior midline of the head and stretching diagonally to the juncture between the eyebrow-occipital line and the anterior border of the corner of the temporal hairline, which is indistinct. Draw a vertical line upwards from the middle point of the zygomatic arch to the eyebrow-occipital line; the intersection of the two lines is the projection of the motor area. The motor area is divided into five equal parts: the upper one-fifth being the motor area of the lower limbs and the trunk, the middle two-fifths being the motor area of the upper limbs, and the lower two fifths the motor area of the face (Fig. 3 shows the motor area of Jiao’s scalp acupuncture). The motor area of the cerebral infarction lesion’s side is selected as the site for acupuncture treatment.

**Fig. 3:**
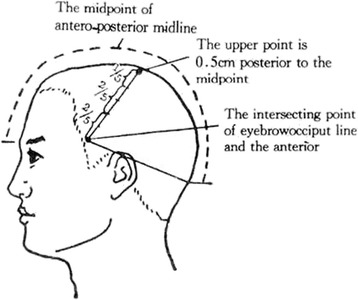
The motor area of Jiao’s scalp acupuncture
2. Acupuncture manipulation: Disposable stainless steel needles (size 0.25 mm × 40 mm, Huatuo brand, manufactured by Suzhou Medical Appliance in Suzhou, Jiangsu Province, China) will be manually inserted at an approximately 15-degree angle to a depth of 1.0–1.5 cm respectively along the upper point and middle point of the motor area on the scalp. For treating motor dysfunction, the needles will be rotated for at least 200 revolutions per minute for 1 minute every 10 minutes for a total of 60 minutes. Scalp acupuncture treatment will be performed by an independent certified practitioner (acupuncturist) with 5 years of clinical experience.
3. Treatment course: The scalp acupuncture treatment will be implemented five times a week, twenty times per treatment course, with each patient having two treatment courses in total.

### Rehabilitation treatment

The patients will receive the conventional rehabilitation programs as mentioned above. The rehabilitation programs will be carried out five times a week (that is, Monday through Friday) for 8 weeks, and every time, the rehabilitation treatment (PT and OT) will last for approximately 1 hour. All rehabilitation treatments will be carried out by qualified therapists.

### Follow-up

After the 8-week treatment observation, all patients will start an additional 8-week follow-up period. Because of the specificity of stroke patients’ recovery, patients from both groups will need to attend community-based rehabilitation treatment during the follow-up period. However, scalp acupuncture treatment is not permitted for stroke patients in both groups during the community-based rehabilitation period. During the 8-week follow-up period, all of the patients from both groups will be reassessed using the FMA, MBI, SSTCM, and SS-QOL at week 12 and week 16 and will be asked to fill out forms to record their rehabilitation treatment attendance. All assessment scales and forms will be returned to the researchers for reviewing at the end of the trial.

### Outcome measures

Data collection will be performed by a trained assessor who is blind to patients’ assignment at baseline, after the intervention (4 weeks, 8 weeks), and at the end of follow-up (12 weeks, 16 weeks).

### Basic characteristic variables

All of the participants’ general status demographic information such as age, sex, educational background, marital status, working condition, and time since attack will be attained from baseline questionnaires. Vital signs (pulse, respiration rate, temperature, and blood pressure) will be measured by nurses.

### Primary outcome measurement

#### Fugl-Meyer Assessment (FMA)

The FMA scale for motor function was developed as the first quantitative evaluative instrument for measuring sensorimotor stroke recovery, which includes an assessment of the upper extremities (UE, 33 items) and lower extremities (LE, 17 items) [31]. The FMA scale includes flexor synergy, extensor synergy, movement combining synergies, movement out of synergy, wrist, hand, and coordination/speed. The motor FM assessments are scored on a 3-point ordinal scale (0–2). Each item can be divided into three levels, the lowest level being 0 point, the highest being 2 points, and level between the two indicated with 1 point. The FM motor assessment is used to measure voluntary limb movement. It includes the UE subscale (33 items; score range 0–66) and the LE subscale (17 items; score range 0–34) for a total motor FM score of 100 [32].

The assessment is performed in a quiet area when the patient is maximally alert. The motor domain has well-established reliability and validity as an indicator of motor impairment severity across different stroke recovery time points [33, 34]. The clinical value of the FM assessment is that it provides a hierarchical scale of motor impairment severity; low FM scores indicate greater impairment. A higher FM score for the UE or LE is a clinical indicator of less motor impairment [35, 36]. The minimal clinically important difference (MCID) values [37–39] of the Chinese version of the FMA in motor domain in patients with stroke are 4.58 for UE, 3.31 for LE, and 6.0 for UE plus LE [40]. We set up a standardized procedure for the FM motor assessments, an adequate training program, and a competency assessment for study raters to ensure rater competence across the duration of the trial. The FMA will be assessed at baseline, during the interventions period (at 4 weeks and 8 weeks), and during the follow-up period (at 12 weeks and 16 weeks).

### Secondary outcome measures

#### Modified Barthel Index (MBI)

The Barthel Index is a scale that measures ten basic aspects of daily life activities related to self-care and mobility [41]. For the Chinese MBI version, the ten items are continence of bowels and bladder, feeding, dressing, entering and leaving a toilet, grooming, bathing, moving from a wheelchair to a bed and returning to a wheelchair, walking on a level surface for 45 m, and ascending and descending stairs. The standards for evaluation are as follows. Each item (activity) can be divided into five levels; each level represents a different degree of independence, the lowest level being 1 and the highest being 5, and the higher the level, the greater the independence. The normal score is 100. If a person’s score is 100, he is able to get along without attendant care. The MBI will be assessed at baseline, during the interventional period (at 4 weeks and 8 weeks), and during the follow-up period (at 12 weeks and 16 weeks).

#### Stroke-Specific Quality of Life (SS-QOL) scale

The SS-QOL is a patient-reported outcome measure intended to provide an assessment of health-related quality of life, specific to patients with stroke [42]. The SS-QOL questionnaire consists of 49 items in the 12 domains of energy, family roles, language, mobility, mood, personality, self-care, social roles, thinking, upper extremity function, vision, and work. Scoring on the SS-QOL is rated on a 5-point Likert scale. Response options are scored as 5 (“no help needed/no trouble at all/strongly disagree”), 4 (“a little help/a little trouble/moderately disagree”), 3 (“some help/some trouble/neither agree nor disagree”), 2 (“a lot of help/a lot of trouble/moderately agree”), and 1 (“total help/could not do it at all/strongly agree”). The domains are scored separately; a total score is also calculated, with higher scores indicating better function. The SS-QOL will be assessed at baseline, during the interventional period (at 4 weeks and 8 weeks), and during the follow-up period (at 12 weeks and 16 weeks).

#### Stroke Syndrome of TCM (SSTCM)

The SSTCM was developed mainly based on a quantified index of TCM symptoms. The SSTCM includes signs and symptoms which cause the most concern for patients and doctors after stroke. SSTCM mainly consists of two domains: TCM symptoms, and pulse conditions and tongue pictures. The TCM symptoms area contains 24 items. The assessment standards of each item are divided into four levels and corresponding scores (normal = 0, light = 1, middle = 2, heavy = 3), based on the severity of the symptoms and their impact on life. Pulse conditions and tongue pictures are simply recorded and have no impact on the score. The total score is calculated using only the first domain, with lower scores indicating a lighter degree of symptom severity and a less significant impact on life [43, 44]. Experienced doctors of TCM who accepted the unification of the assessment training evaluated the SSTCM. The SSTCM will be assessed at baseline, during the interventional period (at 4 weeks and 8 weeks), and during the follow-up period (at 12 weeks and 16 weeks).

### Safety

We will conduct the following tests on all participants at the screening stage to exclude patients with serious organic lesions: white blood cells, platelets, hemoglobin, alanine aminotransferase/aspartate aminotransferase, gamma-glutamyl transpeptidase, creatinine, and blood urea nitrogen.

The subjects will be requested to report information about adverse events (AEs). All AEs that occur during the trial period will be recorded, such as sweating, pallor, dizziness, fainting, perturbed or chest congestion during acupuncture treatment, local hematoma, bleeding, unbearable prickling, local anaphylaxis, retained needle after treatment, and continuous severe local pain for more than one hour after acupuncture. The researcher will confirm the occurrence of AEs and record all details such as the time of occurrence, date, degree, measurement related to the acupuncture treatment, and causal relationship with the acupuncture treatment. Serious AEs must be reported to the principal investigator immediately.

### Quality control

Before the trial, all staff members are required to attend a series of training sessions. These sessions will ensure that the personnel involved fully understand the research protocol and standard operating procedures for the study. To maintain the clinical trial at a consistently high quality, the clinical research center of Longhua Hospital will monitor the study file, informed consent forms, case report forms (CRFs), serious AEs, and data records regularly.

### Data collection, management, and monitoring

The CRF, Treatment Form, and Adverse Events Form will be first completed and then double-entered into the electronic data capture (EDC) system electronically by two independent investigators to act as the first level of control to ensure the accuracy of the data. The second level of data integrity will include data monitoring and validation, which will be conducted on a regular basis throughout the study. The original CRFs and all other forms (including the consent forms) will be archived securely in the clinical research center of Longhua Hospital, affiliated with the Shanghai University of TCM for 5 years following publication of the last paper or report from the study.

The safety of the study will be monitored by a Data and Safety Monitoring Board (DSMB) of the clinical evaluation center of Longhua Hospital, affiliated with Shanghai University of TCM, which consists of independent clinical experts and statisticians with access to unblinded data. The DSMB is independent from the sponsor, the competing interests, and the investigational site and will review the performance and safety of the trial monthly.

The criteria for unblinding and discontinuing allocated interventions for a given trial participant include having a recurrent stroke, having serious complications of stroke or experiencing serious acupuncture-related AEs (if any), which have been described previously. The DSMB will reveal a participant’s allocated intervention and make the final decision to terminate the trial.

The final trial data set will be under the custody of Longhua Hospital/Shanghai University of TCM. The data manager from the clinical evaluation center of Longhua Hospital will have access to the complete, anonymous final data set. Access to the final data set or identifiable data by others will require written requests to be approved by the DSMB of the clinical evaluation center of Longhua Hospital/Shanghai University of TCM and all study investigators.

### Sample size calculation

The sample size was determined using the results of our previous clinical trial and pilot trial [45–47]. The primary efficacy parameter is the change in FMA scores from baseline to the end of treatment after 8 weeks. According to our preliminary test and previous study, the primary efficacy parameter (FMA score) of the control group (rehabilitation treatment) would be increased by 9.86, and that of the treatment group (scalp acupuncture combination rehabilitation treatment) would be increased by 19.12. The FMA average standard deviation would be approximately 7.64. A two-sided 5% significance level and 90% power were considered, and the above relevant data (9.86, 19.12, 7.64, α = 0.05, 1 – β = 0.9) were input into NCSS-PASS V11.0.7 software (https://www.ncss.com/software/pass/) [48]. On the basis of the software calculation, approximately 45 participants are required in each group in order to have a sufficient sample size. With an estimated dropout rate of 20%, each group is required to have 54 initial participants.

### Participant recruitment

Participants will be recruited from two hospitals (Longhua Hospital affiliated with the Shanghai University of Traditional Chinese Medicine and the Shanghai Changning District Tianshan Traditional Chinese Medicine Hospital, which is a Longhua Hospital branch) in Shanghai, China. Our study will be propagated via the Internet, local health-related newspapers, and posters in communities and hospitals. Prospective participants will be asked to talk face to face with study coordinators to discuss the study and provide information regarding eligibility criteria. If patients are eligible and interested in participating, they will be invited for a series of rehabilitation assessments after diagnosis by neurologists. One hundred and eight patients will be included in the study. When their informed consent has been obtained, patients will be randomized into two groups with different treatments.

### Randomization and allocation concealment

Prior to treatment, each patient will be randomly assigned one serial number using a software program. Assignments will be sealed in opaque envelopes and will be opened by the researchers following informed consent procedures and baseline testing. All rehabilitation therapists, assessors, and analysts will be blinded to group assignments.

### Statistical analysis

All data will be analyzed in the clinical research center of Longhua Hospital, affiliated with the Shanghai University of TCM by statisticians using the Statistical Product and Service Solutions (SPSS ) statistical package program (version 17.0, SPSS Inc., Chicago, IL, USA). Baseline assessments will be conducted before randomization, including gender and age of patients, disease course, hemiplegia (left or right), stroke risk factors, diseased location and size, primary outcome (FMA), and secondary outcomes (MBI, SS-QOL, and SSTCM). All patients randomized to each group are included in the analysis, and the data analysis will be conducted using two-sided significance tests at a 5% significance level. All analyses will be based on the intention-to-treat principle using the last observation carried forward rule. Missing values will be handled by the mixed model for repeated measurements. Continuous variables with normal distribution will be expressed as means with standard deviations (SDs) and compared by an independent sample Student *t* test. For abnormally distributed variables, the data will be expressed as medians with ranges, and non-parametric tests will be used. Categorical variables will be expressed as number (%) and analyzed by *χ* ^2^ test or Fisher’s exact test. Descriptive statistics will be used to detail baseline participant demographics and general status characteristics of patients, such as gender, age, disease course, hemiplegia (left or right), stroke risk factors, and diseased location and size. Repeated measures analysis of variance (ANOVA) will be used to analyze value changes of FMA, MBI, SS-QOL, and SSTCM scores across five testing time points (weeks 0, 4, 8, 12, and 16). Safety analyses will be compared with the incidence of AEs in the two groups using the *χ* ^2^ test.

## Discussion

Chinese scalp acupuncture is a contemporary acupuncture technique integrating traditional Chinese needling methods with western medical knowledge of representative areas of the cerebral cortex. As acupuncture was developing, various physicians began to introduce western neurophysiology into the field of acupuncture and explored correlations between the brain and human body. Dr. Jiao Shun-fa, who is the founder of Jiao’s scalp acupuncture and a neurosurgeon in Shan Xi Province, is also the recognized founder of Chinese scalp acupuncture. Dr. Jiao combined the modern understanding of neurophysiology and neuroanatomy with the traditional concept of acupuncture to develop the new scalp acupuncture utilized to affect the functions of the central nervous system. Scalp acupuncture uses special techniques to harmonize and regulate the functional activities of the brain and body. Many researches on scalp acupuncture have indicated positive results in treating various disorders of the central nervous system. Stroke is one of the most common diseases for which acupuncture treatment is recommended, according to the WHO [49]. In view of the motor dysfunction that occurs post-stroke, the upper one-fifth and middle two-fifths of the motor area on the scalp were selected as the primary area for the scalp acupuncture stimulation region.

In stroke rehabilitation clinical studies, rehabilitation evaluation plays an important role. The FMA and MBI were selected as the gold standards for the evaluation of acupuncture treatment’s curative effect in almost all acupuncture treatment stroke researches, both domestically and overseas. We used the FMA scale to best observe the comprehensive motor ability of stroke patients based on the specific environments and the corresponding instructions; the FMA reflects the motor dysfunction level of stroke patients and is widely applied in the evaluation of motor dysfunction after stroke [50]. The MBI was used as an individual-level assessment scale to evaluate daily life activities of stroke patients; it responds to the complex activity ability and necessary functional skills of patients in daily environments and is often used to assess the degree of influence of motor dysfunction in families and social environments [51]. However, in Chinese medicine, stroke itself is thought to involve several interpromoting disease mechanisms, possibly including qi stagnation, heat, phlegm, blood stasis, and wind. Hence, the common name for stroke in Chinese medicine is wind stroke. According to the “four diagnostic methods” of TCM, the syndrome changes in stroke patients are in a dynamic development process. These symptoms’ academic terminologies are not relevant to the understanding of western traditional medicine for the symptoms of stroke diagnosis, including hemiplegia, aphasia, and facial nerve paralysis, etc. [52]. Many motor function assessment scales do not accurately reflect the actual curative effect of acupuncture based on syndrome differentiation of TCM in a certain stage of stroke. The study of acupuncture treatment of stroke should fully reflect and embody the characteristics of TCM [53]. It needs to develop the evaluation standard of acupuncture’s curative effect based on TCM symptomatology. It is not conducive to reasonably judge the effectiveness of acupuncture intervention based on the priority of “syndrome differentiation” because of the lack of an appropriate therapeutic effect evaluation standard of syndromes. However, SSTCM in this research conforms to the TCM identification rule of similar syndromes and highlights the advantages and characteristics of TCM [54, 55].

Acupuncture is a frequently used therapy for stroke rehabilitation in China, but the evidence of its effect from previous studies seems to be inconclusive [56]. Some systematic reviews have been done to study the effect of acupuncture on stroke rehabilitation [14, 25, 57, 58]. These reviews have drawn consistent conclusions that acupuncture appears to be safe and effective for stroke rehabilitation, but the benefits require further confirmation with larger, more transparent and well-conducted randomized clinical trials. Thus, the purpose of this research is to observe the therapeutic effect of scalp acupuncture using Jiao’s motor area for motor dysfunction in patients with ischemic stroke according to both international general evaluation scales and TCM evaluation systems.

Under strict quality control, this study could potentially confirm whether or not scalp acupuncture on a motor area is an effective adjunct to the standard rehabilitation treatment for stroke patients with motor dysfunction. This study also aims to explore the correlation between the TCM symptoms improved and the motor function recovery for patients with stroke, which is of great significance in further improving the evaluation system of acupuncture treatment on motor dysfunction in these patients.

## Trial status

This study has been completed. 

## Additional file

Additional file 1: SPIRIT checklist. (DOC 127 kb)

## Abbreviations

CG: Control group
CRF: Case report form
DSMB: Data and Safety Monitoring Board
EDC: Electronic data capture
FMA: Fugl-Meyer Assessment
LE: Lower extremities
MBI: Modified Barthel Index
MCID: Minimal clinically important difference
MMSE: Mini-Mental State Examination
NIH: National Institutes of Health
RCT: Randomized controlled trial
SS-QOL: Stroke-Specific Quality of Life (scale)
SSTCM: Stroke Syndrome of Traditional Chinese Medicine
TCM: Traditional Chinese medicine
TG: Treatment group
UE: Upper extremities
